# Supplementary material for: How soon should patients be eligible for differentiated service delivery models for antiretroviral treatment? Evidence from a retrospective cohort study in Zambia
Source: BMJ Open. 2022 Dec 21;12(12):e064070. doi: 10.1136/bmjopen-2022-064070 (PMC9772670; doi:10.1136/bmjopen-2022-064070)
Supplement: Author's manuscript [file bmjopen-2022-064070.draft_revisions.pdf]

BMJ Open is committed to open peer review. As part of this commitment we make the peer review history of every article we publish publicly available.

When an article is published we post the peer reviewers' comments and the authors' responses online. We also post the versions of the paper that were used during peer review. These are the versions that the peer review comments apply to.

The versions of the paper that follow are the versions that were submitted during the peer review process. They are not the versions of record or the final published versions. They should not be cited or distributed as the published version of this manuscript.

BMJ Open is an open access journal and the full, final, typeset and author-corrected version of record of the manuscript is available on our site with no access controls, subscription charges or pay-per-view fees (http://bmjopen.bmj.com).

If you have any questions on BMJ Open's open peer review process please email info.bmjopen@bmj.com

BMJ Open

How soon should patients be eligible for differentiated service delivery models for antiretroviral treatment? Evidence from Zambia

Journal:	*BMJ Open*
Manuscript ID	bmjopen-2022-064070
Article Type:	Original research
Date Submitted by the Author:	25-Apr-2022
Complete List of Authors:	Jamieson, Lise; University of the Witwatersrand Faculty of Health Sciences, Health Economics and Epidemiology Research Office<br>Rosen, Sydney; Boston University School of Public Health, Department of Global Health; Health Economics and Epidemiology Research Office, Department of Internal Medicine, Faculty of Health Sciences, University of the Witwatersrand<br>Phiri, Bevis; Clinton Health Access Initiative<br>Grimsrud, Anna; International AIDS Society<br>Mwansa, Muya; Ministry of Health<br>Shakwelele, Hilda; Clinton Health Access Initiative<br>Haimbe, Prudence; Clinton Health Access Initiative<br>Mukumbwa-Mwenechanya, Mpande; Center for Infectious Disease Research in Zambia, Implementation Science Unit<br>Lumano-Mulenga, Priscilla; Ministry of Health<br>Chiboma, Innocent; Ministry of Health<br>Nichols, Brooke ; Health Economics and Epidemiology Research Office; Boston University School of Public Health, Department of Global Health
Keywords:	Health policy < HEALTH SERVICES ADMINISTRATION & MANAGEMENT, HIV & AIDS < INFECTIOUS DISEASES, Public health < INFECTIOUS DISEASES

SCHOLARONE™
Manuscripts

I, the Submitting Author has the right to grant and does grant on behalf of all authors of the Work (as defined in the below author licence), an exclusive licence and/or a non-exclusive licence for contributions from authors who are: i) UK Crown employees; ii) where BMJ has agreed a CC-BY licence shall apply, and/or iii) in accordance with the terms applicable for US Federal Government officers or employees acting as part of their official duties; on a worldwide, perpetual, irrevocable, royalty-free basis to BMJ Publishing Group Ltd ("BMJ") its licensees and where the relevant Journal is co-owned by BMJ to the co-owners of the Journal, to publish the Work in this journal and any other BMJ products and to exploit all rights, as set out in our licence.

The Submitting Author accepts and understands that any supply made under these terms is made by BMJ to the Submitting Author unless you are acting as an employee on behalf of your employer or a postgraduate student of an affiliated institution which is paying any applicable article publishing charge ("APC") for Open Access articles. Where the Submitting Author wishes to make the Work available on an Open Access basis (and intends to pay the relevant APC), the terms of reuse of such Open Access shall be governed by a Creative Commons licence – details of these licences and which Creative Commons licence will apply to this Work are set out in our licence referred to above.

Other than as permitted in any relevant BMJ Author's Self Archiving Policies, I confirm this Work has not been accepted for publication elsewhere, is not being considered for publication elsewhere and does not duplicate material already published. I confirm all authors consent to publication of this Work and authorise the granting of this licence.

For peer review only

How soon should patients be eligible for differentiated service delivery models for antiretroviral treatment? Evidence from Zambia

Short title: Outcomes of early DSD enrolment

Lise Jamieson[1,2], Sydney Rosen[1,3], Bevis Phiri[4], Anna Grimsrud[5], Muya Mwansa[6], Hilda Shakwelele[4], Prudence Haimbe[4], Mpande M Mwenechanya[7], Priscilla Lumano-Mulenga[6], Innocent Chiboma[6], Brooke E Nichols[1,2,3*]

[1]Health Economics and Epidemiology Research Office (HE²RO), Department of Internal Medicine, Faculty of Health Sciences, University of the Witwatersrand, Johannesburg, South Africa

[2]Department of Medical Microbiology, Amsterdam University Medical Centre, Amsterdam, the Netherlands

[3]Department of Global Health, Boston University School of Public Health, Boston, MA, USA

[4]Clinton Health Access Initiative, Lusaka, Zambia

[5]International AIDS Society, Cape Town, South Africa

[6]Ministry of Health, Lusaka, Zambia

[7]The Centre for Infectious Disease Research in Zambia, Lusaka, Zambia

*Corresponding author: Brooke Nichols (brooken@bu.edu); Department of Global Health, Boston University School of Public Health, 801 Massachusetts Avenue, Boston, MA, USA.Tel:+1 857-544-7250

Keywords: differentiated service delivery (DSD) models, HIV, antiretroviral treatment, retention in care, differentiated service delivery guidelines, Zambia

Word count:

Abstract: 300/300

Manuscript: 2,719/5,000

For peer review only - http://bmjopen.bmj.com/site/about/guidelines.xhtml

ABSTRACT

Objectives: Patient attrition is high the first 6 months after antiretroviral therapy (ART) initiation. Patients with <6 months ART are systematically excluded from most differentiated service delivery (DSD) models, which are intended to reduce attrition. Despite DSD eligibility criteria requiring ≥6 months on ART, some patients enroll earlier. We compared loss to follow-up (LTFU) between patients enrolling in DSD models early to those enrolled according to guidelines, assessing whether the ART experience eligibility criterion is necessary.

Setting: In a retrospective cohort study using routinely-collected electronic medical record data in Zambia, we assessed adults (≥15 years) who initiated ART between 01/01/2019 and 31/12/2020, evaluating LTFU (>30 days late for scheduled visit) at 18 months for "early enrollers" (DSD enrolment after <6 months on ART) and "established enrollers" (DSD enrolment after ≥6 months on ART). We used a log-binomial model to compare LTFU risk, adjusting for age, sex, location, ART refill interval, DSD model.

Participants: For 6,340 early enrollers and 25,857 established enrollers there were no differences in sex (61% female), age (median 37 years), or location (65% urban). ART refill intervals were longer for established vs early enrollers (72% vs 55% were given 4–6-month refills).

Results: LTFU at 18 months was 3% (192/6,340) for early enrollers and 5% (24,646/25,857) for established enrollers. Early enrollers were 41% less likely to be LTFU than established patients (adjusted risk ratio [95% confidence interval] 0.59 [0.50-0.68]).

Conclusions: Patients enrolled in DSD after <6 months' ART were more likely to be retained than patients established on ART prior to DSD enrolment. A limitation is that early enrollers may have been selected for DSD due to providers' and patients' expectations about future retention. Offering DSD models to at least some ART patients soon after ART initiation may help address high attrition during the early treatment period.

For peer review only

KEY QUESTIONS

What is already known on this topic

- Differentiated service delivery (DSD) for HIV treatment can increase access and remove barriers to care.
- DSD models are generally designed for patients who are established in care, having at least 6 months of treatment before being eligible for DSD model enrolment.
- Studies have shown that patients in DSD treatment models in sub-Saharan Africa (SSA) have a similar retention in care (generally within 5%), compared to patients who access treatment in conventional, facility-based care.

What this study adds

- Limited data exists on patient outcomes of those who enrol in DSD models early, i.e. patients with <6 months of HIV treatment.
- We show that patients in Zambia who enrolled into DSD models, designed for established patients, early were significantly less likely to be lost to follow-up compared to patients who enrolled into DSD models as per guideline criteria.

How this study might affect research, practice or policy

- This analysis provides a critical first step towards the reassessment of the delayed DSD enrolment policies.
- This work signals that further research needs to be conducted in other SSA countries to evaluate patient outcomes for early DSD model enrolment.

For peer review only

INTRODUCTION

A critical step toward achieving universal coverage of antiretroviral therapy (ART) for HIV is to support lifelong patient retention in ART programmes. Data from sub-Saharan Africa (SSA), where some 70% of the world's ART patients reside, continue to indicate insufficient retention on ART,[1] with about a fifth of all patients lost to care five years after treatment initiation.[2] A patient's first six months after initiation are a high risk period for attrition: a Zambian study showed rates of loss to follow-up to be four-fold higher in the first six months of ART treatment compared to the period between six months and 3.5 years thereafter.[3]

Since 2016, the World Health Organization (WHO) has recommended differentiated service delivery (DSD) for HIV treatment.[4] DSD models such as facility-based individual "fast track" medication pickup and community-based ART refills can increase access and remove barriers to care by adjusting the cadre of provider, location of service delivery, frequency of interactions with the healthcare system, and/or types of services offered to support long-term retention of people established on HIV treatment.[5] A recent systematic review reporting on outcomes of patients in DSD models in SSA found that retention in care of those in DSD models was generally within 5% of that for conventional care.[6] In Zambia, several DSD models have shown to have similar rates of retention as conventional care 12 months after DSD model entry.[7,8] The INTERVAL trial, a cluster-randomized, non-inferiority trial conducted in Malawi and Zambia, found that 6-month ART dispensing was non-inferior in terms of 12-month retention, compared to standard of care.[8] DSD models have consistently been found to save substantial time and money for patients themselves, and satisfaction with the models among both providers and patients has been high.[8–10]

A major limitation of DSD models to date has been eligibility criteria that limit model enrollment to patients on the standard first-line ART regimen who are "stable" or "established on treatment," defined as having been on ART for at least 6 or 12 months and having documented viral suppression.[8,11–13] Until April 2021, the WHO's definition of "established" included at least 12 months of ART experience; new guidelines require at least 6 months on ART for DSD model eligibility [14]. Patients who are newly

For peer review only - http://bmjopen.bmj.com/site/about/guidelines.xhtml

initiated on ART are thus systematically excluded from stable-patient-specific DSD models and from the benefits they offer. In the previously cited INTERVAL trial in Malawi and Zambia, 10% of all patients were excluded due to having initiated ART less than 6 months prior.[15] For patients not eligible for DSD models, guidelines typically require frequent visits to the healthcare facility and medication dispensing intervals of no more than 3 months.[16] In Zambia, all care is differentiated and dependent on the needs of the patient,[11] but currently there is no evidence on the outcomes of patients with <6 months ART experience who enroll into DSD models that are typically reserved for stable patients.

Despite existing guidelines limiting DSD eligibility based on time on ART, in practice patients who do not meet guideline-recommended criteria are sometimes enrolled in DSD models for stable patients, due to provider decision, error or patient request. To begin to understand how such patients who are referred early to DSD models fare when participating in DSD models designed for those established on treatment, we analyzed routinely collected medical record data from Zambia to compare rates of retention among patients enrolled into DSD models earlier than guidelines recommend with retention among those who met all eligibility criteria.

METHODS

Study population and outcomes

We conducted a retrospective cohort study with data extracted in October 2021 from SmartCare, Zambia's national electronic medical record system.[17] We extracted data for patients, aged 15 years or older, reported to have initiated ART between January 2019 and December 2020 at any of 692 health facilities across all 10 provinces. Zambian policy guidelines for this period required patients to be stable on ART before they are considered for DSD enrolment, with stability defined in the 2018 consolidated ART guidelines[11,12] as on ART for at least six months.

We defined patients who enrolled into a DSD model with <6 months of ART as "early enrollers", while a comparison group of patients who enrolled into a DSD model with ≥6 months of ART as "established

For peer review only - http://bmjopen.bmj.com/site/about/guidelines.xhtml

enrollers". Patients on second-line ART (defined as those dispensed protease inhibitors such as lopinavir, atazanavir or ritonavir) were excluded from this analysis, as they are already known to be at high risk of attrition.[18,19] For both early and established enrollers, we assessed loss to follow-up (LTFU) at 18 months post-ART initiation, with LTFU defined as patients who were reported as "lost to follow-up" or "inactive" in the SmartCare database between 15 and 21 months after ART initiation date. "Inactive" was defined as having missed a scheduled visit by more than 30 days. Rates of LTFU were calculated for early and established enrollers and stratified by DSD model type and ART dispensing duration. DSD models, which had multiple names in the SmartCare database, were grouped into the following categories: 1) adherence groups (community adherence groups, rural/urban adherence groups); 2) extended clinic hours (DSD models designed for clinic access before/after hours or weekends, including scholar models); 3) fast-track (procedures to accelerate dispensing at clinics); 4) home ART delivery; 5) multi-month dispensing (MMD); and 6) community pick-up point (central dispensing units, community retail pharmacies, community ART distribution points, health posts, mobile ART distribution models) (Table 1).

Table 1. Differentiated service delivery (DSD) models for HIV treatment in use in Zambia during the study period

Category	Model(s) in category	Description
Adherence groups	Community adherence groups	Patient groups, consisting of ±6 members, meeting at an agreed time every 1-3 months. The groups are managed by the patients themselves, and usually meet outside of the health facility. Members collect ART at clinical appoints for other members in a rotating fashion.[7]
	Rural and urban adherence groups/clubs	Patient groups, consisting of 20-30 members, meeting at an agreed time every 2-3 months. Groups are often facilitated by the same health care worker or facility-based volunteer, also providing pre-packaged ART.[7]
Community pick-up point	Central dispensing units	A centralized model for ART distribution, where medication is packed at a centrally located hub and distributed to patients at multiple approved pick-up points. Clinic visits occur every 6 months at the health facility.[11]

For peer review only - http://bmjopen.bmj.com/site/about/guidelines.xhtml

Category	Model(s) in category	Description
	Community ART distribution points, community retail pharmacies, health posts	ART refills are provided to patients outside of health facilities, e.g. schools, churches, community centres, community retail pharmacies and health posts.[11]
	Mobile ART distribution models	A clinical outreach team linked to a facility does 3-monthly clinical assessments at community distribution points. This model is usually used for hard-to-reach areas.[11]
Extended clinic hours	Before/after-hours models, weekend models, scholar models	These models allow patients to have a clinical visit and collect their ART outside the conventional operation times at the facility (early mornings, evenings and over weekends). These are beneficial to patients with competing priorities (e.g. school or employment).
Fast-track	Fast-track	A model that typically involves a separate, shorter queue to dispense ART to stable patients, allowing for a quick patient visit when a clinical visit is not required.[20]
Home ART delivery	Home ART delivery	Trained community health workers (CHWs) linked to facilities conduct home visits to deliver ART, conduct health screening, monitor adherence, and refer patients as required.[7]
Multi-month dispensing	Multi-month dispensing	Facility-based model in which the primary goal is to dispense medications for more than one month (usually 6 months). Dispensing is typically done during a clinical facility-based visit.

For peer review only

Statistical analysis

We described the demographics of our study population using descriptive statistics. We compared loss to follow-up risk between early enrollers and established enrollers and Wilson's score interval was used to calculate 95% confidence intervals around proportions. We used a log-binomial regression to calculate risk ratios for loss to follow-up, adjusting for age, sex, urban/rural status, DSD model type and ART dispensing duration. Analyses were also stratified by DSD model type and ART dispensing duration. Further, we also conducted an age-stratified analysis and a sub-analysis restricted to facilities with a higher proportion of early enrollers, with results shown in the supplementary material.

For peer review only - http://bmjopen.bmj.com/site/about/guidelines.xhtml

Patient and public involvement

Patients and the public were not involved in the design and conduct of this research.

RESULTS

Study populations

The full SmartCare data set included 1,520,125 unique patients on ART over 2018-2021, of which 32,197 patients had enrolled into a DSD model after ART initiation and had an 18-month outcome reported within the 15-to-21-month window (Figure 1). Of these, 6,340 patients were reported to have been enrolled in DSD models <6 months after ART initiation during the study period (early enrollers). The remaining 25,857 patients comprised the comparison group of established enrollers. For early enrollers, median time enrolled in a DSD model at the time of outcome evaluation was 14.7 months (IQR 13.0-16.5); majority (81%, n=20,856) of established enrollers were on DSD models at outcome evaluation at a median of 5.8 months (interquartile range (IQR) 2.9-8.9) (Table 2). Early enrollers and established enrollers were similar with respect to age, sex and urban/rural location. Across both groups, the median age was 37 years (IQR 29 – 44), a majority (61%, 19,580/32,197) were female and most patients resided in urban settings (64%, n=20,618).

Table 2. Demographics of patients enrolled in differentiated service delivery models

Variable		Early enrollers of DSD models (N=6,340)	Established enrollers of DSD models (N=25,857)
**Age in years, median (IQR)**		36 (29-44)	37 (29-44)
**Age group**	15-24	727 (11%)	2,589 (10%)
	25-34	2,069 (33%)	8,346 (32%)
	35-49	2,658 (42%)	11,424 (44%)
	50+	885 (14%)	3,487 (13%)
**Sex**	Female	3,914 (62%)	15,666 (61%)
	Male	2,426 (38%)	10,191 (39%)
**Location**	Rural	2,501 (39%)	9,078 (35%)
	Urban	3,839 (61%)	16,779 (65%)
**Year of ART initiation**	2019	2,897 (46%)	17,346 (67%)
	2020	3,443 (54%)	8,511 (33%)
**DSD type**	Adherence groups	149 (2%)	508 (2%)
	Community pickup points	671 (11%)	1,461 (6%)
	Extended clinic hours	85 (1%)	97 (<1%)
	Fast-track	979 (15%)	6,266 (24%)

For peer review only

For peer review only - http://bmjopen.bmj.com/site/about/guidelines.xhtml

Variable		Early enrollers of DSD models (N=6,340)	Established enrollers of DSD models (N=25,857)
	Home ART delivery	355 (6%)	973 (4%)
	Multi-month dispensing	4,101 (65%)	16,552 (64%)
ART months dispensed	<2 months	636 (10%)	1,476 (6%)
	3 months	2,197 (35%)	5,688 (22%)
	4-6 months	3,507 (55%)	18,679 (72%)
Outcome Year	2020	2,863 (45%)	17,283 (67%)
	2021	3,477 (55%)	8,574 (33%)
**Months on ART at outcome, median (IQR)**		17.9 (16.4-19.5)	18.4 (16.7-19.8)
**On DSD at outcome**	Yes	6,340 (100%)	20,856 (81%)
	No	0 (0%)	5,001 (19%)
**Months on DSD at outcome, median (IQR)**		14.7 (13.0-16.5)	5.8 (2.9-8.9)
**Patient outcomes by 18 months after ART initiation**	On treatment	6,133 (97%)	24,646 (95%)
	Died	11 (<1%)	31 (<1%)
	Lost to follow-up	192 (3%)	1,169 (5%)
	Stopped ART	4 (<1%)	10 (<1%)
	Stopped DSD	0 (0%)	1 (<1%)

Most patients were enrolled in either multi-month dispensing DSD models (65% [n=4,101] of early enrollers and 64% [n=16,552] of established enrollers) or fast-track (15% [n=979] of early enrollers and 24% [n=6,266] of established enrollers) (Table 1). Amongst early enrollers, around half (55%, n=3,477) were dispensed 4-6 months of ART at their most recent ART pickup, 35% (n=2,197) were dispensed 3 months of ART, and 10% (n=636) were dispensed <2 months of ART. Established enrollers had slightly longer dispensing intervals with 72% (n=18,679) dispensed 4-6 months of ART, 22% (n=5,688) dispensed 3 months of ART, and 6% (n=1,476) dispensed <2 months of ART (Table 1).

Outcomes

Early enrollers had a slightly lower rate of loss to follow-up (3.0% [95% confidence interval (CI) 2.6%-3.5%]) compared to the established enrollers (4.5% [4.3%-4.8%]) (Table 3). Early enrollers experienced similar or lower loss to follow-up rates than established enrollers across nearly all differentiated models of care. The exception was extended clinic hours: early enrollers enrolled in the extended clinic hours model had a similar rate of loss to follow-up than established enrollers (10.6%; [5.7%-18.9%] vs. 8.2% [4.2%-15.4%], respectively). Across both early and established enrollers, longer dispensing periods were associated with lower rates of loss to follow-up, which increased from 2.5%-3.8% for 4-6-month

For peer review only

For peer review only - http://bmjopen.bmj.com/site/about/guidelines.xhtml

dispensing to 3.5%-5.3% for 3-month dispensing to 4.1%-10.6% for <2-month dispensing (Table 3). Early enrollers with <2 months dispensing had a lower rate of loss to follow-up than did established enrollers (4.1%; [2.8%-5.9%] vs. 10.6% [9.1%-12.2%]).

Table 3. Relative risk of loss to follow-up at 18 months post-ART initiation for early enrollers of differentiated service delivery (DSD) models

	Proportion of patients lost to follow-up at 18 months, % (95% CI) [n/N]			
	**Early enrollers**	**Established enrollers**	**Unadjusted risk ratio (95% CI)**	**Adjusted risk ratio* (95% CI)**
**All patients**	3.0% (2.6% - 3.5%) [192/6,340]	4.5% (4.3% - 4.8%) [1,169/25,857]	0.67 (0.57-0.78)	0.59 (0.50-0.68)
**Stratification: DSD model**				
Adherence groups	2.7% (1% - 6.7%) [4/149]	3.1% (1.9% - 5.1%) [16/508]	0.85 (0.25-2.29)	0.79 (0.23-2.12)
Community pickup points	4.5% (3.1% - 6.3%) [30/671]	3.3% (2.5% - 4.3%) [48/1,461]	1.36 (0.86-2.12)	1.30 (0.81-2.03)
Extended clinic hours	10.6% (5.7% - 18.9%) [9/85]	8.2% (4.2% - 15.4%) [8/97]	1.28 (0.51-3.27)	1.19 (0.43-3.34)
Fast track	3.4% (2.4% - 4.7%) [33/979]	3.6% (3.2% - 4.1%) [227/6,266]	0.93 (0.64-1.31)	0.74 (0.50-1.05)
Home ART delivery	1.4% (0.6% - 3.3%) [5/355]	6.3% (4.9% - 8%) [61/973]	0.22 (0.08-0.50)	0.18 (0.06-0.41)
Multi-month dispensing	2.7% (2.3% - 3.2%) [111/4,101]	4.9% (4.6% - 5.2%) [809/16,552]	0.55 (0.45-0.67)	0.51 (0.41-0.61)
**Stratification: ART dispensing duration**				
<2 months	4.1% (2.8% - 5.9%) [26/636]	10.6% (9.1% - 12.2%) [156/1,476]	0.39 (0.25-0.57)	0.40 (0.26-0.59)
3 months	3.5% (2.8% - 4.4%) [77/2,197]	5.3% (4.8% - 5.9%) [303/5,688]	0.66 (0.51-0.84)	0.64 (0.49-0.81)
4-6 months	2.5% (2.1% - 3.1%) [89/3,507]	3.8% (3.5% - 4.1%) [709/18,679]	0.67 (0.54-0.83)	0.67 (0.53-0.82)

*Model adjusted for age, sex, location, ART dispensing duration and DSD model type

In an analysis adjusting for age, sex, location, ART dispensing duration, and DSD model type, early enrollers in all DSD model types and dispensing durations were 41% less likely to be lost to follow-up than established enrollers (adjusted risk ratio (aRR) 0.59 [0.50-0.68]) (Table 3). The reduced adjusted risk of being lost to follow-up were similar for patients in adherence groups (aRR 0.79 [0.23-2.12]), multi-month dispensing (aRR 0.51 [0.41-0.61]), home ART delivery (aRR 0.18 [0.06-0.41]) and fast track models (aRR 0.74 [0.50-1.05]). Early enrollers had a statistically insignificant increased risk of being lost to follow-up in the community pick-up point (aRR 1.30 [0.81-2.03]) and extended clinic hours models (aRR 1.19 [0.43-3.34]) compared to the established enrollers.

For peer review only - http://bmjopen.bmj.com/site/about/guidelines.xhtml

An age-stratified analysis produced similar results to the main analysis, with early enrollers in each age group being less likely to be lost to follow-up than established enrollers in the same age group. However, the effect of earlier enrollment in DSD on reduced loss to follow-up appeared less pronounced in patients on 4-6 months' ART dispensing for those aged 25 to 49 years (Appendix Figure S1). In facilities where a larger proportion of all DSD patients enrolled in DSD models early, the trend towards early enrollers performing better persisted with respect to loss to follow-up compared to outcomes for established enrollers (Appendix Figure S2).

DISCUSSION

In nearly all of sub-Saharan Africa, DSD model eligibility criteria require that patients be on ART for a minimum of six months (and in some countries a minimum of 12 months) prior to DSD model enrollment.[21] We present novel data from Zambia highlighting good outcomes when newly initiated ART patients (those with less than 6 months' ART experience) are referred early to DSD models. Those referred early to DSD appear to have good outcomes across different DSD models and age categories.

Our data begin to fill in a gap in the evidence base on the validity of time on treatment as an eligibility criterion for DSD models. Because few if any countries permit DSD model enrollment for new initiators, little evidence on their experience in DSD models has been available until now. To date, most reports on DSD outcomes have been limited to people who have spent a significant amount of time on ART prior to DSD model enrollment. In the previously mentioned INTERVAL trial, for example, participants had been on ART for a median of roughly five years at DSD model entry, while patients in a trial of multi-month dispensing in adherence clubs in South Africa had a median duration on ART of 7.3 years at baseline.[22]

While ART patients in Zambia have historically been lost to follow-up at high rates in the first few months after ART initiation,[3] in our DSD patient population this was less likely to be the case. Our results provide evidence to support the recent revision of WHO guidelines that reduce time on ART from 12 to six months on treatment as part the definition of "established" on ART.[14] These findings

For peer review only

offer reassurance and evidence to countries that have expanded eligibility as they scale up DSD models,[21,23] particularly to support uninterrupted access to HIV treatment during the COVID-19 pandemic, that earlier referral to DSD is possible without compromising patient care. Even if many, or most, of the patients in our "early enrollment" sample were selected deliberately because they were considered at low loss to follow-up risk, our results demonstrate that early eligibility for DSD models should be considered for at least some patients before they reach six months on ART.

Loss to follow up at 18 months after ART initiation for early and established enrollers averaged 1-11% for all six categories of DSD models studied. We did not observe any programmatically important differences by model or ART experience prior to model enrollment. Where a programmatically important difference did arise, in contrast, was in dispensing intervals. Regardless of how long a patient had been on ART at DSD model enrollment, patients who received ≤2 months of medications at a time were more likely to be lost to follow up than patients who received either 3 months or 4-6 months of medications. This likely reflects providers' assessments of patients' ability to remain on treatment and/or clinical condition. Those regarded as being at higher risk of attrition are asked to come to the clinic for medication refills more often, so that they can be monitored and supported more closely. Ironically, difficulty in accessing the clinic may be the very reason that some patients are at high risk of attrition. For these patients, insisting on shorter refill durations may simply exacerbate whatever challenges they face.

There were several limitations to our analysis. First, as noted above, we assume that patients with <6 months on ART in our sample were not offered DSD model enrollment at random. If providers made accurate clinical decisions about individual patients' risks of attrition, patients in our "early enrollment" cohorts could over-represent patients thought to have low attrition risk. To achieve the results we found, providers would have had to make these decisions correctly at multiple sites across the entire country. If this is the case, our data suggest that the healthcare workers responsible for enrolling patients into DSD models can successfully identify those who will do well with early enrollment. At the same time, if the early enrollers in our data set do comprise patients at lower risk of loss to follow-up, then our

For peer review only - http://bmjopen.bmj.com/site/about/guidelines.xhtml

results likely underestimate the true rate of loss to follow-up that would occur if early DSD enrollment were to be broadly available, without the benefit of provider selection.

A second limitation is that our data set included only patients reported in the electronic medical record system to have enrolled in a DSD model. It is possible that some patients not in DSD models may be recorded as enrolled, and some who were enrolled may have been missed. Third, bias could occur if facilities with better-than-average retention in care were also more likely to allow early DSD model enrollment. In this case, our results may reflect differences in facility quality, as well as enrollment timing. An analysis restricted to facilities with >20% early DSD enrolment showed an even lower risk of loss to follow-up among patients enrolled early into DSD models, however, compared to patients with >6 months of ART at DSD entry.

Despite these limitations, our analysis demonstrates that patients on ART for less than six months who are enrolled in existing DSD models can be successfully retained in care and may even fare better than those left in conventional care and only initiate DSD models greater than six months after ART initiation. It is likely that not all patients are ready for less intensive DSD models in their first half-year or year on treatment, but some clearly are. Since DSD models have been shown to be beneficial to patients and in some cases to providers, offering enrollment to newly-initiating ART patients may improve ART programs in general. Future research should look more closely at which patients can be enrolled early and which models of care serve these patients best.

CONCLUSION

Current policy for DSD model eligibility criteria in Zambia, as in other countries, have required a minimum of 12 months of ART before a patient is considered for DSD enrolment, and more recently, a minimum of six months of ART. In order to change guidelines to allow DSD enrolment sooner after ART initiation (i.e., 6 months or less), large-scale observational evidence, implementation research or trial data demonstrating good patient outcomes among those who enrol in DSD models < six months' post ART initiation would be required. This analysis therefore provides a critical first step towards the

For peer review only - http://bmjopen.bmj.com/site/about/guidelines.xhtml

reassessment of the delayed DSD enrolment policies, and signals that further research needs to be conducted in other SSA countries to evaluate patient outcomes for early DSD model enrolment.

COMPETING INTERESTS

We declare no competing interests.

AUTHORS' CONTRIBUTIONS

LJ, BN, SR and AG conceptualized the study. BP, HS, PH, MM, PLM, IC curated data for the study. BP, HS, PH, MMM provided supervision of the study. LJ led data analysis and drafted the paper along with BN, SR and AG. All authors contributed to data interpretation and critically reviewed a revised draft of the manuscript. All authors have read and approved the final manuscript.

FUNDING

Funding for the study was provided by the Bill & Melinda Gates Foundation through OPP1192640 to Boston University. The funder had no role in study design, data collection and analysis, decision to publish or preparation of the manuscript.

DATA SHARING

The data is owned by the Zambian Ministry of Health and the use of it was approved by the Human Research Ethics Committee (University of Witwatersrand, Johannesburg, South Africa) and ERES Converge IRB (Zambia). All relevant data is included in the paper and supplementary material. The full data are available upon approval from Zambian Ministry of Health and appropriate ethics committees.

ETHICS

This study protocol was approved by ERES Converge IRB (Zambia), protocol number 2019-Sep-030, the Human Research Ethics Committee (Medical) of the University of Witwatersrand, protocol number M190453, and the Boston University IRB H-38823 for the use of data with a waiver of consent.

For peer review only

REFERENCES

1. *UNAIDS AIDSinfo: People Living with HIV Receiving ART*.; 2020. Accessed June 15, 2021. https://aidsinfo.unaids.org/

2. Haas AD, Zaniewski E, Anderegg N, et al. Retention and mortality on antiretroviral therapy in sub-Saharan Africa: collaborative analyses of HIV treatment programmes. *J Intern AIDS Soc*. 2018;21(2):e25084. doi:10.1002/jia2.25084

3. Schöni-Affolter F, Keiser O, Mwango A, et al. Estimating Loss to Follow-Up in HIV-Infected Patients on Antiretroviral Therapy: The Effect of the Competing Risk of Death in Zambia and Switzerland. Myer L, ed. *PLoS ONE*. 2011;6(12):e27919. doi:10.1371/journal.pone.0027919

4. *World Health Organization. Guidelines on HIV Self-Testing and Partner Notification, Supplement to Consolidated Guidelines on HIV Testing Services*.; 2016. Accessed November 18, 2020. https://www.who.int/hiv/pub/vct/hiv-self-testing-guidelines/en/

5. Duncombe C, Rosenblum S, Hellmann N, et al. Reframing HIV care: putting people at the centre of antiretroviral delivery. *Trop Med Int Health*. 2015;20(4):430-447. doi:10.1111/tmi.12460

6. Long L, Kuchukhidze S, Pascoe S, et al. Retention in care and viral suppression in differentiated service delivery models for HIV treatment delivery in sub-Saharan Africa: a rapid systematic review. *J Intern AIDS Soc*. 2020;23(11). doi:10.1002/jia2.25640

7. Nichols BE, Cele R, Jamieson L, et al. Community-based delivery of HIV treatment in Zambia: costs and outcomes. *AIDS*. 2021;35(2):299-306. doi:10.1097/QAD.0000000000002737

8. Hoffman RM, Moyo C, Balakasi KT, et al. Multimonth dispensing of up to 6 months of antiretroviral therapy in Malawi and Zambia (INTERVAL): a cluster-randomised, non-blinded, non-inferiority trial. *The Lancet Global Health*. 2021;9(5):e628-e638. doi:10.1016/S2214-109X(21)00039-5

9. Nichols BE, Cele R, Lekodeba N, et al. Economic evaluation of differentiated service delivery models for HIV treatment in Lesotho: costs to providers and patients. *J Intern AIDS Soc*. 2021;24(4). doi:10.1002/jia2.25692

10. Guthrie T, Muheki C, Greener R, et al. *Costs and Outcomes of Differentiated ART Service Delivery in Uganda: Summary of Findings*.; 2020. https://sites.bu.edu/ambit/files/2021/02/Uganda-EQUIP-Brief-ART-DSDM-cost-outcomes-FINAL-2020.08.24.pdf

11. *Ministry of Health. Zambia Differentiated Service Delivery Framework*.; 2018.

12. *Republic of Zambia. Ministry of Health. Zambia Consolidated Guidelines for Treatment and Prevention of HIV Infection*.; 2018.

13. *Republic of Zambia. Ministry of Health. Zambia Consolidated Guidelines for Treatment and Prevention of HIV Infection*.; 2020.

14. *World Health Organization. Updated Recommendations on Service Delivery for the Treatment and Care of People Living with HIV*.; 2021. https://apps.who.int/iris/rest/bitstreams/1344311/retrieve

For peer review only - http://bmjopen.bmj.com/site/about/guidelines.xhtml

15. Hoffman RM, Balakasi K, Bardon AR, et al. Eligibility for differentiated models of HIV treatment service delivery: an estimate from Malawi and Zambia. *AIDS*. 2020;34(3):475-479. doi:10.1097/QAD.0000000000002435

16. Rosen S, Grimsrud A, Ehrenkranz P, Katz I. Models of service delivery for optimizing a patient's first six months on antiretroviral therapy for HIV: an applied research agenda. *Gates Open Res*. 2020;4:116. doi:10.12688/gatesopenres.13159.1

17. Gumede-Moyo S, Todd J, Bond V, Mee P, Filteau S. A qualitative inquiry into implementing an electronic health record system (SmartCare) for prevention of mother-to-child transmission data in Zambia: a retrospective study. *BMJ Open*. 2019;9(9):e030428. doi:10.1136/bmjopen-2019-030428

18. Wandeler G, Keiser O, Mulenga L, et al. Tenofovir in Second-Line ART in Zambia and South Africa: Collaborative Analysis of Cohort Studies. *JAIDS Journal of Acquired Immune Deficiency Syndromes*. 2012;61(1):41-48. doi:10.1097/QAI.0b013e3182632540

19. Kebede HK, Mwanri L, Ward P, Gesesew HA. Predictors of lost to follow up from antiretroviral therapy among adults in sub-Saharan Africa: a systematic review and meta-analysis. *Infect Dis Poverty*. 2021;10(1):33. doi:10.1186/s40249-021-00822-7

20. Huber A, Pascoe S, Nichols B, et al. Differentiated Service Delivery Models for HIV Treatment in Malawi, South Africa, and Zambia: A Landscape Analysis. *Glob Health Sci Pract*. Published online May 10, 2021:ghsp;GHSP-D-20-00532v1. doi:10.9745/GHSP-D-20-00532

21. *Time on ART before Eligibility for DSD for HIV Treatment. Differentiated Service Delivery.* International AIDS Society (IAS); 2020. https://differentiatedservicedelivery.org/Portals/0/adam/Content/jcdklT8RzEqirRdlckAjbQ/File/1-Time%20to%20DSD%20Eligibility%20D5.pdf

22. Cassidy T, Grimsrud A, Keene C, et al. Twenty-four-month outcomes from a cluster-randomized controlled trial of extending antiretroviral therapy refills in ART adherence clubs. *J Int AIDS Soc*. 2020;23(12):e25649. doi:10.1002/jia2.25649

23. Grimsrud A, Wilkinson L. Acceleration of differentiated service delivery for HIV treatment in sub-Saharan Africa during COVID-19. *J Int AIDS Soc*. 2021;24(6). doi:10.1002/jia2.25704

Figure 1: Flow diagram depicting study population

For peer review only - http://bmjopen.bmj.com/site/about/guidelines.xhtml

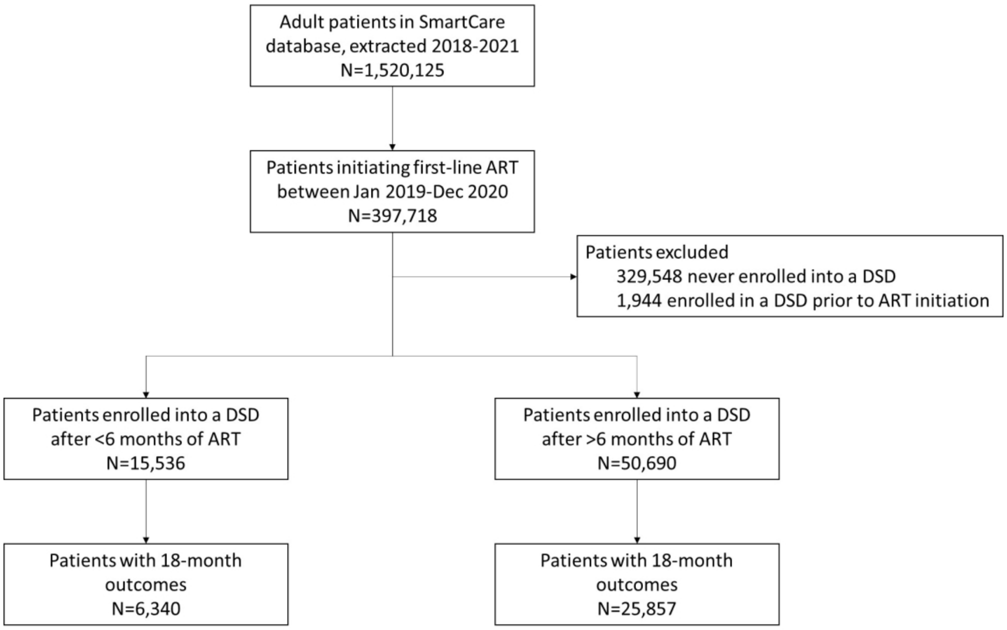

Flow diagram depicting study population

157x99mm (300 x 300 DPI)

Supplementary Appendix to:

How soon should patients be eligible for differentiated service delivery models for antiretroviral treatment?

Lise Jamieson[1,2*], Sydney Rosen[1,3], Bevis Phiri[4], Anna Grimsrud[5], Muya Mwansa[6], Hilda Shakwelele[4], Prudence Haimbe[4], Mpande M Mwenechanya[7], Priscilla Lumano Mulenga[6], Innocent Chiboma[6], Brooke E Nichols[1,2,3*]

[1]Health Economics and Epidemiology Research Office (HE[2]RO), Department of Internal Medicine, Faculty of Health Sciences, University of the Witwatersrand, Johannesburg, South Africa; [2]Department of Medical Microbiology, Amsterdam University Medical Centre, Amsterdam, the Netherlands; [3]Department of Global Health, Boston University School of Public Health, Boston, MA, USA; [4]Clinton Health Access Initiative, Lusaka, Zambia; [5]International AIDS Society, Cape Town, South Africa; [6]Ministry of Health, Lusaka, Zambia [7]The Centre for Infectious Disease Research in Zambia, Lusaka, Zambia

Figure S1. Relative risk of loss to follow-up within 18 months of ART initiation for early enrollers of DSD models (ie. after <6 months of ART), stratified by dispensing period and age group (reference group: established enrollers of DSD models with >6 months of ART at DSD enrolment; analysis adjusted for sex and urban/rural status)

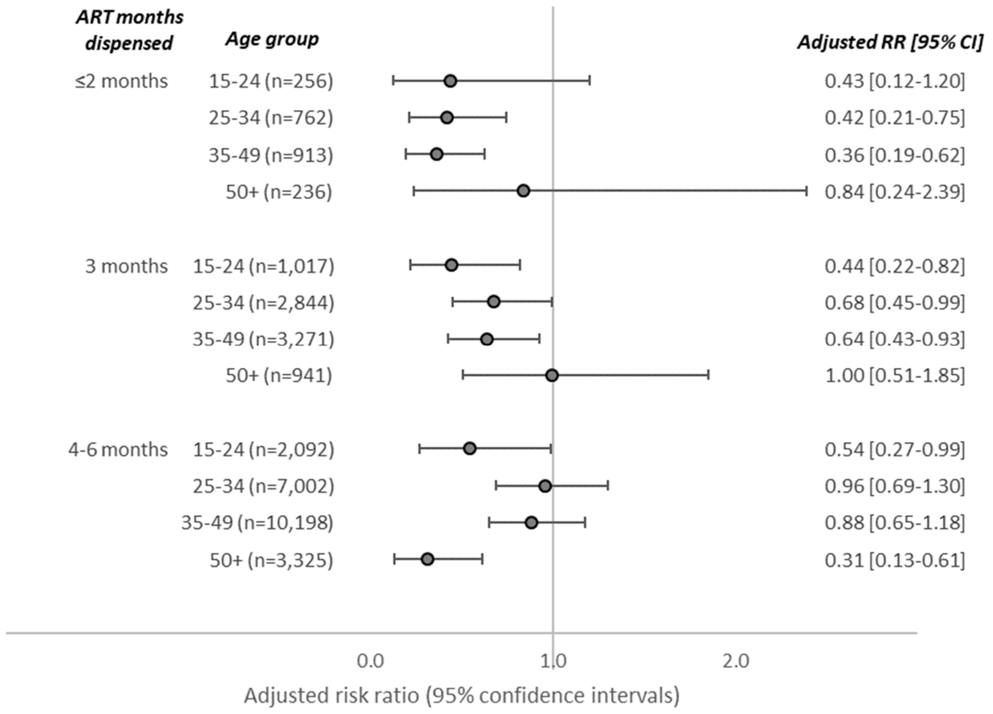

For peer review only - http://bmjopen.bmj.com/site/about/guidelines.xhtml

Figure S2. Proportion loss to follow-up by time on ART at DSD entry, limited to N=37 facilities with >20% of DSD patients at each facility classified as "early enrollers"

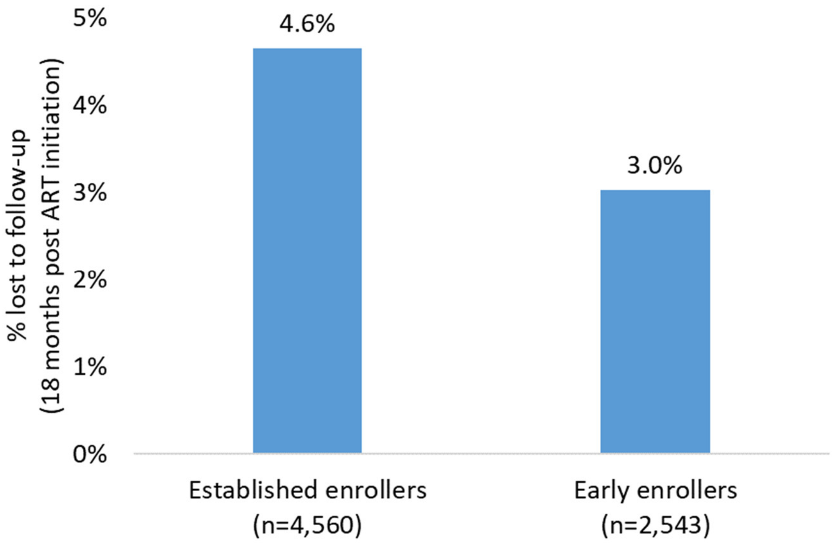

A potential area of concern was that facilities that had better-than-average retention would be more willing or able to enroll patients into DSD models early and therefore skew the results. We therefore conducted this sub-analysis where we limited the data to those facilities which had substantial proportion of their patients enrolled into DSD models early. Criteria for this analysis limited the data to facilities where: i) ≥20% of patients had early enrollers, and ii) at least 100 patients across both groups (early enrollers and established enrollers). 37 facilities across 8 of 10 provinces were selected for this analysis; 73% (n=27) of facilities were in urban areas. This analysis consisted of 7,103 patients: majority (61%, n=4,351) were female, age group distribution was similar to the main analysis (Table 2) (11%, n=784 were 15-24 years; 35%, n=2,488 were 25-34 years; 43%, n=3,028 were 35-49 years; 11%, n=799 were 50+ years), 81% (n=5,731) of patients were in urban settings. Majority (57%, n=4,028) of patients were enrolled into multi-month dispensing, 29% (n=2,058) were in fast-track, 7% (n=484) were in community pick-up points, 5% (n=350) were in home ART delivery, and <2% were in adherence groups (n=112) and extended clinic hours' groups (n=71).

Results show that in this subset of clinics, early enrollers were less likely to be lost to follow-up (3.0% [77/2,543]), compared to established enrollers (4.6% [212/4,560]). A log-binomial regression assessing risk of loss to follow-up, adjusting for age, sex, urban/rural status, and ART dispensing period estimated that, compared to established enrollers, early enrollers were 40% less likely to be lost to follow-up; adjusted risk ratios (aRR) 0.60 (95% CI 0.46-0.78).

For peer review only - http://bmjopen.bmj.com/site/about/guidelines.xhtml

STROBE Statement—Checklist of items that should be included in reports of ***cohort studies***

	Item No	Recommendation	Page No
**Title and abstract**	1	(*a*) Indicate the study's design with a commonly used term in the title or the abstract<br>(*b*) Provide in the abstract an informative and balanced summary of what was done and what was found	2
**Introduction**			
Background/rationale	2	Explain the scientific background and rationale for the investigation being reported	4-5
Objectives	3	State specific objectives, including any prespecified hypotheses	5
**Methods**			
Study design	4	Present key elements of study design early in the paper	5-6
Setting	5	Describe the setting, locations, and relevant dates, including periods of recruitment, exposure, follow-up, and data collection	5-6
Participants	6	(*a*) Give the eligibility criteria, and the sources and methods of selection of participants. Describe methods of follow-up<br>(*b*) For matched studies, give matching criteria and number of exposed and unexposed	5-6
Variables	7	Clearly define all outcomes, exposures, predictors, potential confounders, and effect modifiers. Give diagnostic criteria, if applicable	5-6
Data sources/ measurement	8*	For each variable of interest, give sources of data and details of methods of assessment (measurement). Describe comparability of assessment methods if there is more than one group	5
Bias	9	Describe any efforts to address potential sources of bias	5-6
Study size	10	Explain how the study size was arrived at	5-6
Quantitative variables	11	Explain how quantitative variables were handled in the analyses. If applicable, describe which groupings were chosen and why	5-7
Statistical methods	12	(*a*) Describe all statistical methods, including those used to control for confounding<br>(*b*) Describe any methods used to examine subgroups and interactions<br>(*c*) Explain how missing data were addressed<br>(*d*) If applicable, explain how loss to follow-up was addressed<br>(*e*) Describe any sensitivity analyses	7
**Results**			
Participants	13*	(a) Report numbers of individuals at each stage of study—eg numbers potentially eligible, examined for eligibility, confirmed eligible, included in the study, completing follow-up, and analysed<br>(b) Give reasons for non-participation at each stage<br>(c) Consider use of a flow diagram	8, Figure 1
Descriptive data	14*	(a) Give characteristics of study participants (eg demographic, clinical, social) and information on exposures and potential confounders<br>(b) Indicate number of participants with missing data for each variable of interest<br>(c) Summarise follow-up time (eg, average and total amount)	8-9, Table 2
Outcome data	15*	Report numbers of outcome events or summary measures over time	9-10

For peer review only

Main results	16	(*a*) Give unadjusted estimates and, if applicable, confounder-adjusted estimates and their precision (eg, 95% confidence interval). Make clear which confounders were adjusted for and why they were included<br>(*b*) Report category boundaries when continuous variables were categorized<br>(*c*) If relevant, consider translating estimates of relative risk into absolute risk for a meaningful time period	10
Other analyses	17	Report other analyses done—eg analyses of subgroups and interactions, and sensitivity analyses	11
**Discussion**			
Key results	18	Summarise key results with reference to study objectives	11
Limitations	19	Discuss limitations of the study, taking into account sources of potential bias or imprecision. Discuss both direction and magnitude of any potential bias	12-13
Interpretation	20	Give a cautious overall interpretation of results considering objectives, limitations, multiplicity of analyses, results from similar studies, and other relevant evidence	12-13
Generalisability	21	Discuss the generalisability (external validity) of the study results	13-14
**Other information**			
Funding	22	Give the source of funding and the role of the funders for the present study and, if applicable, for the original study on which the present article is based	14

*Give information separately for exposed and unexposed groups.

Note: An Explanation and Elaboration article discusses each checklist item and gives methodological background and published examples of transparent reporting. The STROBE checklist is best used in conjunction with this article (freely available on the Web sites of PLoS Medicine at http://www.plosmedicine.org/, Annals of Internal Medicine at http://www.annals.org/, and Epidemiology at http://www.epidem.com/). Information on the STROBE Initiative is available at http://www.strobe-statement.org.

For peer review only

BMJ Open

How soon should patients be eligible for differentiated service delivery models for antiretroviral treatment? Evidence from a retrospective cohort study in Zambia

Journal:	*BMJ Open*
Manuscript ID	bmjopen-2022-064070.R1
Article Type:	Original research
Date Submitted by the Author:	03-Nov-2022

Complete List of Authors:	Jamieson, Lise; University of the Witwatersrand Faculty of Health Sciences, Health Economics and Epidemiology Research Office<br>Rosen, Sydney; Boston University School of Public Health, Department of Global Health; Health Economics and Epidemiology Research Office, Department of Internal Medicine, Faculty of Health Sciences, University of the Witwatersrand<br>Phiri, Bevis; Clinton Health Access Initiative<br>Grimsrud, Anna; International AIDS Society<br>Mwansa, Muya; Ministry of Health<br>Shakwelele, Hilda; Clinton Health Access Initiative<br>Haimbe, Prudence; Clinton Health Access Initiative<br>Mukumbwa-Mwenechanya, Mpande; Center for Infectious Disease Research in Zambia, Implementation Science Unit<br>Lumano-Mulenga, Priscilla; Ministry of Health<br>Chiboma, Innocent; Ministry of Health<br>Nichols, Brooke ; Health Economics and Epidemiology Research Office; Boston University School of Public Health, Department of Global Health

**Primary Subject Heading**:	Public health
Secondary Subject Heading:	Global health, Health policy, HIV/AIDS
Keywords:	Health policy < HEALTH SERVICES ADMINISTRATION & MANAGEMENT, HIV & AIDS < INFECTIOUS DISEASES, Public health < INFECTIOUS DISEASES

SCHOLARONE™ Manuscripts

I, the Submitting Author has the right to grant and does grant on behalf of all authors of the Work (as defined in the below author licence), an exclusive licence and/or a non-exclusive licence for contributions from authors who are: i) UK Crown employees; ii) where BMJ has agreed a CC-BY licence shall apply, and/or iii) in accordance with the terms applicable for US Federal Government officers or employees acting as part of their official duties; on a worldwide, perpetual, irrevocable, royalty-free basis to BMJ Publishing Group Ltd ("BMJ") its licensees and where the relevant Journal is co-owned by BMJ to the co-owners of the Journal, to publish the Work in this journal and any other BMJ products and to exploit all rights, as set out in our licence.

The Submitting Author accepts and understands that any supply made under these terms is made by BMJ to the Submitting Author unless you are acting as an employee on behalf of your employer or a postgraduate student of an affiliated institution which is paying any applicable article publishing charge ("APC") for Open Access articles. Where the Submitting Author wishes to make the Work available on an Open Access basis (and intends to pay the relevant APC), the terms of reuse of such Open Access shall be governed by a Creative Commons licence – details of these licences and which Creative Commons licence will apply to this Work are set out in our licence referred to above.

Other than as permitted in any relevant BMJ Author's Self Archiving Policies, I confirm this Work has not been accepted for publication elsewhere, is not being considered for publication elsewhere and does not duplicate material already published. I confirm all authors consent to publication of this Work and authorise the granting of this licence.

For peer review only

How soon should patients be eligible for differentiated service delivery models for antiretroviral treatment? Evidence from a retrospective cohort study in Zambia

Short title: Outcomes of early DSD enrolment

Lise Jamieson[1,2], Sydney Rosen[1,3*], Bevis Phiri[4], Anna Grimsrud[5], Muya Mwansa[6], Hilda Shakwelele[4], Prudence Haimbe[4], Mpande M Mwenechanya[7], Priscilla Lumano-Mulenga[6], Innocent Chiboma[6], Brooke E Nichols[1,2,3]

[1]Health Economics and Epidemiology Research Office (HE[2]RO), Department of Internal Medicine, Faculty of Health Sciences, University of the Witwatersrand, Johannesburg, South Africa

[2]Department of Medical Microbiology, Amsterdam University Medical Centre, Amsterdam, the Netherlands

[3]Department of Global Health, Boston University School of Public Health, Boston, MA, USA

[4]Clinton Health Access Initiative, Lusaka, Zambia

[5]IAS - International AIDS Society, Cape Town, South Africa

[6]Ministry of Health, Lusaka, Zambia

[7]The Centre for Infectious Disease Research in Zambia, Lusaka, Zambia

*Corresponding author: Sydney Rosen (sbrosen@bu.edu); Department of Global Health, Boston University School of Public Health, 801 Massachusetts Avenue, Boston, MA, USA.Tel:+1 857-207-7909

Keywords: differentiated service delivery (DSD) models, HIV, antiretroviral treatment, retention in care, differentiated service delivery guidelines, Zambia

Word count:

Abstract: 300/300

Manuscript: 2,719/5,000

For peer review only

ABSTRACT

Objectives: Patient attrition is high the first six months after antiretroviral therapy (ART) initiation. Patients with <6 months ART are systematically excluded from most differentiated service delivery (DSD) models, which are intended to support retention. Despite DSD eligibility criteria requiring ≥6 months on ART, some patients enroll earlier. We compared loss to follow-up (LTFU) between patients enrolling in DSD models early to those enrolled according to guidelines, assessing whether the ART experience eligibility criterion is necessary.

Setting: In a retrospective cohort study using routinely-collected electronic medical record data in Zambia, we assessed adults (≥15 years) who initiated ART between 01/01/2019 and 31/12/2020, evaluating LTFU (>30 days late for scheduled visit) at 18 months for “early enrollers” (DSD enrolment after <6 months on ART) and “established enrollers” (DSD enrolment after ≥6 months on ART). We used a log-binomial model to compare LTFU risk, adjusting for age, sex, location, ART refill interval, DSD model.

Participants: For 6,340 early enrollers and 25,857 established enrollers there were no differences in sex (61% female), age (median 37 years), or location (65% urban). ART refill intervals were longer for established vs early enrollers (72% vs 55% were given 4–6-month refills).

Results: LTFU at 18 months was 3% (192/6,340) for early enrollers and 5% (24,646/25,857) for established enrollers. Early enrollers were 41% less likely to be LTFU than established patients (adjusted risk ratio [95% confidence interval] 0.59 [0.50-0.68]).

Conclusions: Patients enrolled in DSD after <6 months’ ART were more likely to be retained than patients established on ART prior to DSD enrolment. A limitation is that early enrollers may have been selected for DSD due to providers’ and patients’ expectations about future retention. Offering DSD models to ART patients soon after ART initiation may help address high attrition during the early treatment period.

For peer review only - http://bmjopen.bmj.com/site/about/guidelines.xhtml

STRENGTHS AND LIMITATIONS

- Our analysis utilized data from Zambia's national electronic medical record system, with records from the entire national HIV treatment cohort over four years (2018-2021) in all ten provinces.
- We report observed outcomes for more than 6,000 antiretroviral treatment (ART) clients who enrolled in differentiated service delivery (DSD) models after less than six months' experience on ART.
- Results reflect large-scale, routine program implementation, rather than clinical trial settings.
- A key limitation is the assumption that patients who were enrolled in DSD models after less than 6 months on ART were selected based on an expectation of good future adherence.
- A further limitation is the potential bias if facilities with better-than-average retention rates were more likely to allow early DSD model enrollment; results may reflect differences in the quality of services as opposed to the relationship between duration on ART before DSD enrollment and retention in care.

For peer review only

INTRODUCTION

A critical step toward achieving universal coverage of antiretroviral therapy (ART) for HIV is to support lifelong patient retention in ART programmes. Data from sub-Saharan Africa (SSA), where some 70% of the world's ART patients reside, continue to indicate insufficient retention on ART,[1] with about a fifth of all patients lost to care five years after treatment initiation.[2] A patient's first six months after initiation are a high risk period for attrition: a Zambian study showed rates of loss to follow-up to be four-fold higher in the first six months of ART treatment compared to the period between six months and 3.5 years thereafter.[3]

Since 2016, the World Health Organization (WHO) has recommended differentiated service delivery (DSD) for HIV treatment.[4] DSD models such as facility-based individual "fast track" medication pickup and community-based ART refills can increase access and remove barriers to care by adjusting the cadre of provider, location of service delivery, frequency of interactions with the healthcare system, and/or types of services offered to support long-term retention of people established on HIV treatment.[5] A recent systematic review reporting on outcomes of patients in DSD models in SSA found that retention in care of those in DSD models was generally within 5% of that for conventional care.[6] In Zambia, several DSD models have shown to have similar rates of retention as conventional care 12 months after DSD model entry.[7,8] The INTERVAL trial, a cluster-randomized, non-inferiority trial conducted in Malawi and Zambia, found that 6-month ART dispensing was non-inferior in terms of 12-month retention, compared to standard of care.[8] DSD models have consistently been found to save substantial time and money for patients themselves, and satisfaction with the models among both providers and patients has been high.[8–10]

A major limitation to the scale-up of DSD models to date has been eligibility criteria that limit enrollment to patients who are "stable" or "established on treatment, which is defined as patients who: i) are on first-line ART regimens; ii) have been on ART for at least 6 or 12 months; and iii) have a recent, documented suppressed viral load.[8,11–13] Until April 2021, the WHO's definition of "established" included at least 12 months of ART experience; new guidelines require at least 6 months on ART for

For peer review only - http://bmjopen.bmj.com/site/about/guidelines.xhtml

DSD model eligibility [14]. Patients who are newly initiated on ART are thus systematically excluded from stable-patient-specific DSD models and from the benefits they offer. In the previously cited INTERVAL trial in Malawi and Zambia, 10% of all patients were excluded due to having initiated ART less than 6 months prior.[15] For patients not eligible for DSD models, guidelines typically require frequent visits to the healthcare facility and medication dispensing intervals of no more than 3 months.[16] In Zambia, all care is differentiated and dependent on the needs of the patient,[11] but currently there is no evidence on the outcomes of patients with <6 months ART experience who enroll into DSD models that are typically reserved for stable patients.

Despite existing guidelines limiting DSD eligibility based on time on ART, in practice patients who do not meet guideline-recommended criteria are sometimes enrolled in DSD models for stable patients, due to provider decision, error or patient request. To understand how such patients who are referred early to DSD models fare when participating in DSD models designed for those established on treatment, we analyzed routinely collected medical record data from Zambia to compare rates of retention among patients enrolled into DSD models earlier than guidelines recommend with retention among those who met all eligibility criteria.

METHODS

Study population and outcomes

We conducted a retrospective cohort study with data extracted in October 2021 from SmartCare, Zambia's national electronic medical record system.[17] We extracted data for patients, aged 15 years or older, reported to have initiated ART between January 2019 and December 2020 at any of 692 health facilities across all 10 provinces. Zambian policy guidelines for this period required patients to be stable on ART before they are considered for DSD enrolment, with stability defined in the 2018 consolidated ART guidelines[11,12] as on ART for at least six months.

For peer review only - http://bmjopen.bmj.com/site/about/guidelines.xhtml

We defined patients who enrolled into a DSD model with <6 months of ART as "early enrollers", while a comparison group of patients who enrolled into a DSD model with ≥6 months of ART as "established enrollers". Patients on second-line ART (defined as those dispensed protease inhibitors such as lopinavir, atazanavir or ritonavir) were excluded from this analysis, as they are already known to be at high risk of attrition.[18,19] For both early and established enrollers, we assessed loss to follow-up (LTFU) at 18 months post-ART initiation, with LTFU defined as patients who were reported as "lost to follow-up" or "inactive" in the SmartCare database between 15 and 21 months after ART initiation date. "Inactive" was defined as having missed a scheduled visit by more than 30 days. Rates of LTFU were calculated for early and established enrollers and stratified by DSD model type and ART dispensing duration. DSD models, which had multiple names in the SmartCare database, were grouped into the following categories: 1) adherence groups (community adherence groups, rural/urban adherence groups); 2) extended clinic hours (DSD models designed for clinic access before/after hours or weekends, including scholar models); 3) fast-track (procedures to accelerate dispensing at clinics); 4) home ART delivery; 5) multi-month dispensing (MMD); and 6) community pick-up point (central dispensing units, community retail pharmacies, community ART distribution points, health posts, mobile ART distribution models) (Table 1). These six DSD models were defined for our analysis to be mutually exclusive – patients could only be enrolled in a single model.

Table 1. Differentiated service delivery (DSD) models for HIV treatment in use in Zambia during the study period

Category	Model(s) in category	Description
1. Adherence groups	Community adherence groups	Patient groups, consisting of ±6 members, meeting at an agreed time every 1-3 months. The groups are managed by the patients themselves, and usually meet outside of the health facility. Members collect ART at clinical appoints for other members in a rotating fashion.[7]
	Rural and urban adherence groups/clubs	Patient groups, consisting of 20-30 members, meeting at an agreed time every 2-3 months. Groups are often facilitated by the same health care worker or facility-based volunteer, also providing pre-packaged ART.[7]

For peer review only

Category	Model(s) in category	Description
2. Community pick-up point	Central dispensing units	A centralized model for ART distribution, where medication is packed at a centrally located hub and distributed to patients at multiple approved pick-up points. Clinic visits occur every 6 months at the health facility.[11]
	Community ART distribution points, community retail pharmacies, health posts	ART refills are provided to patients outside of health facilities, e.g. schools, churches, community centres, community retail pharmacies and health posts.[11]
	Mobile ART distribution models	A clinical outreach team linked to a facility does 3-monthly clinical assessments at community distribution points. This model is usually used for hard-to-reach areas.[11]
3. Extended clinic hours	Before/after-hours models, weekend models, scholar models	These models allow patients to have a clinical visit and collect their ART outside the conventional operation times at the facility (early mornings, evenings and over weekends). These are beneficial to patients with competing priorities (e.g. school or employment).
4. Fast-track	Fast-track	A model that typically involves a separate, shorter queue to dispense ART to stable patients, allowing for a quick patient visit when a clinical visit is not required.[20]
5. Home ART delivery	Home ART delivery	Trained community health workers (CHWs) linked to facilities conduct home visits to deliver ART, conduct health screening, monitor adherence, and refer patients as required.[7]
6. Multi-month dispensing	Multi-month dispensing	Facility-based model in which the primary goal is to dispense medications for more than one month (usually 6 months). Dispensing is typically done during a clinical facility-based visit.

Statistical analysis

We described the demographics of our study population using descriptive statistics. We compared loss to follow-up risk between early enrollers and established enrollers and Wilson's score interval was used to calculate 95% confidence intervals around proportions. We used a log-binomial regression to calculate risk ratios for loss to follow-up, adjusting for age, sex, urban/rural status, DSD model type and ART dispensing duration. Analyses were also stratified by DSD model type and ART dispensing

For peer review only - http://bmjopen.bmj.com/site/about/guidelines.xhtml

duration. Further, we also conducted an age-stratified analysis and a sub-analysis restricted to facilities with a higher proportion of early enrollers, with results shown in the supplementary material.

Patient and public involvement

Patients and the public were not involved in the design and conduct of this research.

RESULTS

Study populations

The full SmartCare data set included 1,520,125 unique patients on ART over 2018-2021, of whom 32,197 had enrolled into a DSD model after ART initiation and had an 18-month outcome reported within the 15-to-21-month window (Figure 1). Of these, 6,340 patients were reported to have been enrolled in DSD models <6 months after ART initiation during the study period (early enrollers). The remaining 25,857 patients comprised the comparison group of established enrollers. For early enrollers, median time enrolled in a DSD model at the time of outcome evaluation was 14.7 months (IQR 13.0-16.5); majority (81%, n=20,856) of established enrollers were on DSD models at outcome evaluation at a median of 5.8 months (interquartile range (IQR) 2.9-8.9) (Table 2). Early enrollers and established enrollers were similar with respect to age, sex and urban/rural location. Across both groups, the median age was 37 years (IQR 29 – 44), a majority (61%, 19,580/32,197) were female and most patients resided in urban settings (64%, n=20,618).

For peer review only

Table 2. Demographics of patients enrolled in differentiated service delivery models

Variable		Early enrollers of DSD models (N=6,340)	Established enrollers of DSD models (N=25,857)
**Age in years, median (IQR)**		36 (29-44)	37 (29-44)
**Age group**	15-24	727 (11%)	2,589 (10%)
	25-34	2,069 (33%)	8,346 (32%)
	35-49	2,658 (42%)	11,424 (44%)
	50+	885 (14%)	3,487 (13%)
**Sex**	Female	3,914 (62%)	15,666 (61%)
	Male	2,426 (38%)	10,191 (39%)
**Location**	Rural	2,501 (39%)	9,078 (35%)
	Urban	3,839 (61%)	16,779 (65%)
**Year of ART**	2019	2,897 (46%)	17,346 (67%)

For peer review only - http://bmjopen.bmj.com/site/about/guidelines.xhtml

Variable		Early enrollers of DSD models (N=6,340)	Established enrollers of DSD models (N=25,857)
**initiation**	2020	3,443 (54%)	8,511 (33%)
**DSD type**	Adherence groups	149 (2%)	508 (2%)
	Community pickup points	671 (11%)	1,461 (6%)
	Extended clinic hours	85 (1%)	97 (<1%)
	Fast-track	979 (15%)	6,266 (24%)
	Home ART delivery	355 (6%)	973 (4%)
	Multi-month dispensing	4,101 (65%)	16,552 (64%)
**ART months dispensed**	<2 months	636 (10%)	1,476 (6%)
	3 months	2,197 (35%)	5,688 (22%)
	4-6 months	3,507 (55%)	18,679 (72%)
**Outcome Year**	2020	2,863 (45%)	17,283 (67%)
	2021	3,477 (55%)	8,574 (33%)
**Months on ART at outcome, median (IQR)**		17.9 (16.4-19.5)	18.4 (16.7-19.8)
**On DSD at outcome**	Yes	6,340 (100%)	20,856 (81%)
	No	0 (0%)	5,001 (19%)
**Months on DSD at outcome, median (IQR)**		14.7 (13.0-16.5)	5.8 (2.9-8.9)
**Patient outcomes by 18 months after ART initiation**	On treatment	6,133 (97%)	24,646 (95%)
	Died	11 (<1%)	31 (<1%)
	Lost to follow-up	192 (3%)	1,169 (5%)
	Stopped ART	4 (<1%)	10 (<1%)
	Stopped DSD	0 (0%)	1 (<1%)

Most patients were enrolled in either multi-month dispensing DSD models (65% [n=4,101] of early enrollers and 64% [n=16,552] of established enrollers) or fast-track (15% [n=979] of early enrollers and 24% [n=6,266] of established enrollers) (Table 1). Amongst early enrollers, around half (55%, n=3,477) were dispensed 4-6 months of ART at their most recent ART pickup, 35% (n=2,197) were dispensed 3 months of ART, and 10% (n=636) were dispensed <2 months of ART. Established enrollers had slightly longer dispensing intervals with 72% (n=18,679) dispensed 4-6 months of ART, 22% (n=5,688) dispensed 3 months of ART, and 6% (n=1,476) dispensed <2 months of ART (Table 1).

Outcomes

Early enrollers had a slightly lower rate of loss to follow-up (3.0% [95% confidence interval (CI) 2.6%-3.5%]) compared to the established enrollers (4.5% [4.3%-4.8%]) (Table 3). Early enrollers experienced similar or lower loss to follow-up rates than established enrollers across nearly all differentiated models of care. The exception was extended clinic hours: early enrollers enrolled in the extended clinic hours

For peer review only - http://bmjopen.bmj.com/site/about/guidelines.xhtml

model had a similar rate of loss to follow-up as established enrollers (10.6%; [5.7%-18.9%] vs. 8.2% [4.2%-15.4%], respectively). Across both early and established enrollers, longer dispensing periods were associated with lower rates of loss to follow-up, which increased from 2.5%-3.8% for 4-6-month dispensing to 3.5%-5.3% for 3-month dispensing to 4.1%-10.6% for <2-month dispensing (Table 3). Early enrollers with <2 months dispensing had a lower rate of loss to follow-up than did established enrollers (4.1%; [2.8%-5.9%] vs. 10.6% [9.1%-12.2%]).

Table 3. Relative risk of loss to follow-up at 18 months post-ART initiation for early enrollers of differentiated service delivery (DSD) models

	Proportion of patients lost to follow-up at 18 months, % (95% CI) [n/N]			
	**Early enrollers**	**Established enrollers**	**Unadjusted risk ratio (95% CI)**	**Adjusted risk ratio* (95% CI)**
**All patients**	3.0% (2.6% - 3.5%) [192/6,340]	4.5% (4.3% - 4.8%) [1,169/25,857]	0.67 (0.57-0.78)	0.59 (0.50-0.68)
**Stratification: DSD model**				
Adherence groups	2.7% (1% - 6.7%) [4/149]	3.1% (1.9% - 5.1%) [16/508]	0.85 (0.25-2.29)	0.79 (0.23-2.12)
Community pickup points	4.5% (3.1% - 6.3%) [30/671]	3.3% (2.5% - 4.3%) [48/1,461]	1.36 (0.86-2.12)	1.30 (0.81-2.03)
Extended clinic hours	10.6% (5.7% - 18.9%) [9/85]	8.2% (4.2% - 15.4%) [8/97]	1.28 (0.51-3.27)	1.19 (0.43-3.34)
Fast track	3.4% (2.4% - 4.7%) [33/979]	3.6% (3.2% - 4.1%) [227/6,266]	0.93 (0.64-1.31)	0.74 (0.50-1.05)
Home ART delivery	1.4% (0.6% - 3.3%) [5/355]	6.3% (4.9% - 8%) [61/973]	0.22 (0.08-0.50)	0.18 (0.06-0.41)
Multi-month dispensing	2.7% (2.3% - 3.2%) [111/4,101]	4.9% (4.6% - 5.2%) [809/16,552]	0.55 (0.45-0.67)	0.51 (0.41-0.61)
**Stratification: ART dispensing duration**				
<2 months	4.1% (2.8% - 5.9%) [26/636]	10.6% (9.1% - 12.2%) [156/1,476]	0.39 (0.25-0.57)	0.40 (0.26-0.59)
3 months	3.5% (2.8% - 4.4%) [77/2,197]	5.3% (4.8% - 5.9%) [303/5,688]	0.66 (0.51-0.84)	0.64 (0.49-0.81)
4-6 months	2.5% (2.1% - 3.1%) [89/3,507]	3.8% (3.5% - 4.1%) [709/18,679]	0.67 (0.54-0.83)	0.67 (0.53-0.82)

*Model adjusted for age, sex, location, ART dispensing duration and DSD model type

In an analysis adjusting for age, sex, location, ART dispensing duration, and DSD model type, early enrollers in all DSD model types and dispensing durations were 41% less likely to be lost to follow-up than established enrollers (adjusted risk ratio (aRR) 0.59 [0.50-0.68]) (Table 3). The reduced adjusted risk of being lost to follow-up were similar for patients in adherence groups (aRR 0.79 [0.23-2.12]), multi-month dispensing (aRR 0.51 [0.41-0.61]), home ART delivery (aRR 0.18 [0.06-0.41]) and fast track models (aRR 0.74 [0.50-1.05]). Early enrollers had a statistically insignificant increased risk of

For peer review only

being lost to follow-up in the community pick-up point (aRR 1.30 [0.81-2.03]) and extended clinic hours models (aRR 1.19 [0.43-3.34]) compared to the established enrollers.

An age-stratified analysis produced similar results to the main analysis, with early enrollers in each age group being less likely to be lost to follow-up than established enrollers in the same age group. However, the effect of earlier enrollment in DSD on reduced loss to follow-up appeared less pronounced in patients on 4-6 months' ART dispensing for those aged 25 to 49 years (Appendix Figure S1). In facilities where a larger proportion of all DSD patients enrolled in DSD models early, the trend towards early enrollers performing better persisted with respect to loss to follow-up compared to outcomes for established enrollers (Appendix Figure S2).

DISCUSSION

In nearly all of sub-Saharan Africa, DSD model eligibility criteria require that patients be on ART for a minimum of six months (and in some countries a minimum of 12 months) prior to DSD model enrollment.[21] We present a novel analysis from Zambia highlighting good outcomes when newly initiated ART patients (those with less than 6 months' ART experience) are referred early to DSD models. Those referred early to DSD appear to have good outcomes across different DSD models and age categories.

Our data begin to fill in a gap in the evidence base on the validity of time on treatment as an eligibility criterion for DSD models. Because few if any countries permit DSD model enrollment for new initiators, little evidence on their experience in DSD models has been available until now. To date, most reports on DSD outcomes have been limited to people who have spent a significant amount of time on ART prior to DSD model enrollment. In the previously mentioned INTERVAL trial, for example, participants had been on ART for a median of roughly five years at DSD model entry, while patients in a trial of multi-month dispensing in adherence clubs in South Africa had a median duration on ART of 7.3 years at baseline.[22]

For peer review only - http://bmjopen.bmj.com/site/about/guidelines.xhtml

While ART patients in Zambia have historically been lost to follow-up at high rates in the first few months after ART initiation,[3] in our DSD patient population this was less likely to be the case. Our results provide evidence to support the recent revision of WHO guidelines that reduce time on ART from 12 to six months on treatment as part the definition of "established" on ART.[14] These findings offer reassurance and evidence to countries that have expanded eligibility as they scale up DSD models,[21,23] particularly to support uninterrupted access to HIV treatment during the COVID-19 pandemic, that earlier referral to DSD is possible without compromising patient care. Even if many, or most, of the patients in our "early enrollment" sample were selected deliberately because they were considered at low loss to follow-up risk, our results demonstrate that early eligibility for DSD models should be considered for at least some patients before they reach six months on ART.

Loss to follow up at 18 months after ART initiation for early and established enrollers averaged 1-11% for all six categories of DSD models studied. We did not observe any programmatically important differences by model or ART experience prior to model enrollment. Where a programmatically important difference did arise, in contrast, was in dispensing intervals. Regardless of how long a patient had been on ART at DSD model enrollment, patients who received ≤2 months of medications at a time were more likely to be lost to follow up than patients who received either 3 months or 4-6 months of medications. This likely reflects providers' assessments of patients' ability to remain on treatment and/or clinical condition. Those regarded as being at higher risk of attrition are asked to come to the clinic for medication refills more often, so that they can be monitored and supported more closely. Ironically, difficulty in accessing the clinic may be the very reason that some patients are at high risk of attrition. For these patients, insisting on shorter refill durations may simply exacerbate whatever challenges they face.

There were several limitations to our analysis. First, we cannot explain why some patients were enrolled in DSD models before reaching six months on ART. As noted above, we assume that patients with <6 months on ART in our sample were not offered DSD model enrollment at random. If providers made accurate clinical decisions about individual patients' risks of attrition, patients in our "early enrollment"

For peer review only - http://bmjopen.bmj.com/site/about/guidelines.xhtml

cohorts could over-represent patients thought to have low attrition risk. To achieve the results we found, providers would have had to make these decisions correctly at multiple sites across the entire country. If this is the case, our data suggest that the healthcare workers responsible for enrolling patients into DSD models can successfully identify those who will do well with early enrollment. At the same time, if the early enrollers in our data set do comprise patients at lower risk of loss to follow-up, then our results likely underestimate the true rate of loss to follow-up that would occur if early DSD enrollment were to be broadly available, without the benefit of provider selection.

A second limitation is that our data set included only patients reported in the electronic medical record system to have enrolled in a DSD model. It is possible that some patients not in DSD models may be recorded as enrolled, and some who were enrolled may have been missed. Third, bias could occur if facilities with better-than-average retention in care were also more likely to allow early DSD model enrollment. In this case, our results may reflect differences in facility quality, as well as enrollment timing. An analysis restricted to facilities with >20% early DSD enrolment showed an even lower risk of loss to follow-up among patients enrolled early into DSD models, however, compared to patients with >6 months of ART at DSD entry.

Despite these limitations, our analysis demonstrates that patients on ART for less than six months who are enrolled in existing DSD models can be successfully retained in care and may even fare better than those left in conventional care and only initiate DSD models greater than six months after ART initiation. It is likely that not all patients are ready for less intensive DSD models in their first half-year or year on treatment, but some clearly are. Since DSD models have been shown to be beneficial to patients and in some cases to providers, offering enrollment to newly-initiating ART patients may improve ART programs in general. Future research should look more closely at which patients can be enrolled early and which models of care serve these patients best.

CONCLUSION

For peer review only - http://bmjopen.bmj.com/site/about/guidelines.xhtml

For peer review only

Current policy for DSD model eligibility criteria in Zambia, as in other countries, have required a minimum of 12 months of ART before a patient is considered for DSD enrolment, and more recently, a minimum of six months of ART. In order to change guidelines to allow DSD enrolment sooner after ART initiation (i.e., 6 months or less), large-scale observational evidence, implementation research or trial data demonstrating good patient outcomes among those who enrol in DSD models < six months' post ART initiation would be required. This analysis therefore provides a critical first step towards the reassessment of the delayed DSD enrolment policies, and signals that further research needs to be conducted in other SSA countries to evaluate patient outcomes for early DSD model enrolment.

COMPETING INTERESTS

We declare no competing interests.

AUTHORS' CONTRIBUTIONS

LJ, BN, SR and AG conceptualized the study. BP, HS, PH, MM, PLM, IC curated data for the study. BP, HS, PH, MMM provided supervision of the study. LJ led data analysis and drafted the paper along with BN, SR and AG. All authors contributed to data interpretation and critically reviewed a revised draft of the manuscript. All authors have read and approved the final manuscript.

FUNDING

Funding for the study was provided by the Bill & Melinda Gates Foundation through OPP1192640 to Boston University. The funder had no role in study design, data collection and analysis, decision to publish or preparation of the manuscript.

DATA SHARING

The data is owned by the Zambian Ministry of Health and the use of it was approved by the Human Research Ethics Committee (University of Witwatersrand, Johannesburg, South Africa) and ERES Converge IRB (Zambia). All relevant data is included in the paper and supplementary material. The full data are available upon approval from Zambian Ministry of Health and appropriate ethics committees.

For peer review only

ETHICS

This study protocol was approved by ERES Converge IRB (Zambia), protocol number 2019-Sep-030, the Human Research Ethics Committee (Medical) of the University of Witwatersrand, protocol number M190453, and the Boston University IRB H-38823 for the use of data with a waiver of consent.

For peer review only

For peer review only - http://bmjopen.bmj.com/site/about/guidelines.xhtml

REFERENCES

1. *UNAIDS AIDSinfo: People Living with HIV Receiving ART*.; 2020. Accessed June 15, 2021. https://aidsinfo.unaids.org/

2. Haas AD, Zaniewski E, Anderegg N, et al. Retention and mortality on antiretroviral therapy in sub-Saharan Africa: collaborative analyses of HIV treatment programmes. *J Intern AIDS Soc*. 2018;21(2):e25084. doi:10.1002/jia2.25084

3. Schöni-Affolter F, Keiser O, Mwango A, et al. Estimating Loss to Follow-Up in HIV-Infected Patients on Antiretroviral Therapy: The Effect of the Competing Risk of Death in Zambia and Switzerland. Myer L, ed. *PLoS ONE*. 2011;6(12):e27919. doi:10.1371/journal.pone.0027919

4. *World Health Organization. Guidelines on HIV Self-Testing and Partner Notification, Supplement to Consolidated Guidelines on HIV Testing Services*.; 2016. Accessed November 18, 2020. https://www.who.int/hiv/pub/vct/hiv-self-testing-guidelines/en/

5. Duncombe C, Rosenblum S, Hellmann N, et al. Reframing HIV care: putting people at the centre of antiretroviral delivery. *Trop Med Int Health*. 2015;20(4):430-447. doi:10.1111/tmi.12460

6. Long L, Kuchukhidze S, Pascoe S, et al. Retention in care and viral suppression in differentiated service delivery models for HIV treatment delivery in sub-Saharan Africa: a rapid systematic review. *J Intern AIDS Soc*. 2020;23(11). doi:10.1002/jia2.25640

7. Nichols BE, Cele R, Jamieson L, et al. Community-based delivery of HIV treatment in Zambia: costs and outcomes. *AIDS*. 2021;35(2):299-306. doi:10.1097/QAD.0000000000002737

8. Hoffman RM, Moyo C, Balakasi KT, et al. Multimonth dispensing of up to 6 months of antiretroviral therapy in Malawi and Zambia (INTERVAL): a cluster-randomised, non-blinded, non-inferiority trial. *The Lancet Global Health*. 2021;9(5):e628-e638. doi:10.1016/S2214-109X(21)00039-5

9. Nichols BE, Cele R, Lekodeba N, et al. Economic evaluation of differentiated service delivery models for HIV treatment in Lesotho: costs to providers and patients. *J Intern AIDS Soc*. 2021;24(4). doi:10.1002/jia2.25692

10. Guthrie T, Muheki C, Greener R, et al. *Costs and Outcomes of Differentiated ART Service Delivery in Uganda: Summary of Findings*.; 2020. https://sites.bu.edu/ambit/files/2021/02/Uganda-EQUIP-Brief-ART-DSDM-cost-outcomes-FINAL-2020.08.24.pdf

11. *Ministry of Health. Zambia Differentiated Service Delivery Framework*.; 2018.

12. *Republic of Zambia. Ministry of Health. Zambia Consolidated Guidelines for Treatment and Prevention of HIV Infection*.; 2018.

13. *Republic of Zambia. Ministry of Health. Zambia Consolidated Guidelines for Treatment and Prevention of HIV Infection*.; 2020.

14. *World Health Organization. Updated Recommendations on Service Delivery for the Treatment and Care of People Living with HIV*.; 2021. https://apps.who.int/iris/rest/bitstreams/1344311/retrieve

For peer review only - http://bmjopen.bmj.com/site/about/guidelines.xhtml

15. Hoffman RM, Balakasi K, Bardon AR, et al. Eligibility for differentiated models of HIV treatment service delivery: an estimate from Malawi and Zambia. *AIDS*. 2020;34(3):475-479. doi:10.1097/QAD.0000000000002435

16. Rosen S, Grimsrud A, Ehrenkranz P, Katz I. Models of service delivery for optimizing a patient's first six months on antiretroviral therapy for HIV: an applied research agenda. *Gates Open Res*. 2020;4:116. doi:10.12688/gatesopenres.13159.1

17. Gumede-Moyo S, Todd J, Bond V, Mee P, Filteau S. A qualitative inquiry into implementing an electronic health record system (SmartCare) for prevention of mother-to-child transmission data in Zambia: a retrospective study. *BMJ Open*. 2019;9(9):e030428. doi:10.1136/bmjopen-2019-030428

18. Wandeler G, Keiser O, Mulenga L, et al. Tenofovir in Second-Line ART in Zambia and South Africa: Collaborative Analysis of Cohort Studies. *JAIDS Journal of Acquired Immune Deficiency Syndromes*. 2012;61(1):41-48. doi:10.1097/QAI.0b013e3182632540

19. Kebede HK, Mwanri L, Ward P, Gesesew HA. Predictors of lost to follow up from antiretroviral therapy among adults in sub-Saharan Africa: a systematic review and meta-analysis. *Infect Dis Poverty*. 2021;10(1):33. doi:10.1186/s40249-021-00822-7

20. Huber A, Pascoe S, Nichols B, et al. Differentiated Service Delivery Models for HIV Treatment in Malawi, South Africa, and Zambia: A Landscape Analysis. *Glob Health Sci Pract*. Published online May 10, 2021:ghsp;GHSP-D-20-00532v1. doi:10.9745/GHSP-D-20-00532

21. *Time on ART before Eligibility for DSD for HIV Treatment. Differentiated Service Delivery.* International AIDS Society (IAS); 2020. https://differentiatedservicedelivery.org/Portals/0/adam/Content/jcdklT8RzEqirRdlckAjbQ/File/1-Time%20to%20DSD%20Eligibility%20D5.pdf

22. Cassidy T, Grimsrud A, Keene C, et al. Twenty-four-month outcomes from a cluster-randomized controlled trial of extending antiretroviral therapy refills in ART adherence clubs. *J Int AIDS Soc*. 2020;23(12):e25649. doi:10.1002/jia2.25649

23. Grimsrud A, Wilkinson L. Acceleration of differentiated service delivery for HIV treatment in sub-Saharan Africa during COVID-19. *J Int AIDS Soc*. 2021;24(6). doi:10.1002/jia2.25704

Figure 1: Flow diagram depicting study population

For peer review only - http://bmjopen.bmj.com/site/about/guidelines.xhtml

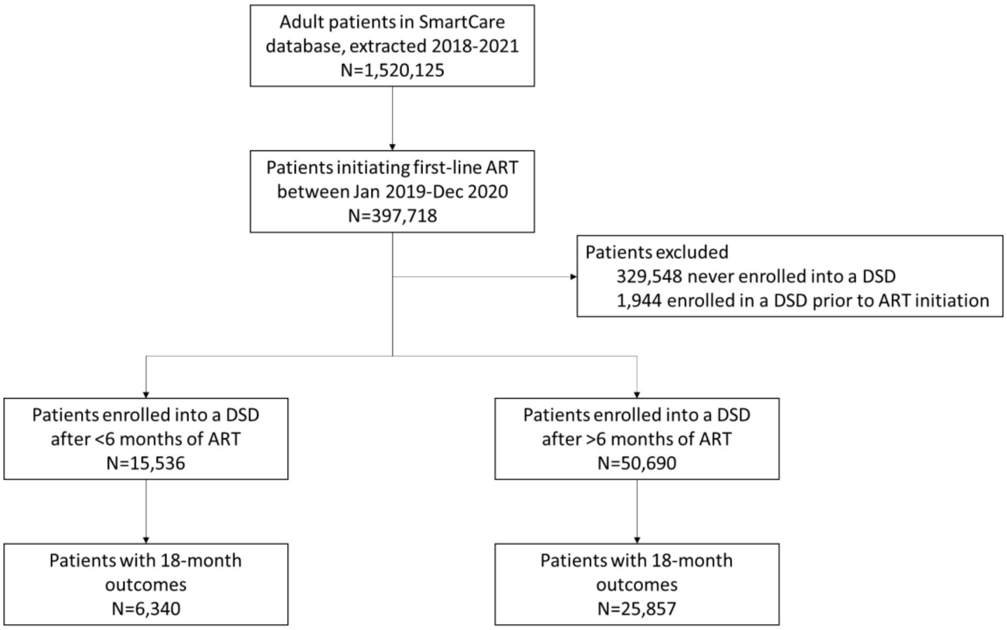

Flow diagram depicting study population

157x99mm (300 x 300 DPI)

Supplementary Appendix to:

How soon should patients be eligible for differentiated service delivery models for antiretroviral treatment?

Lise Jamieson[1,2*], Sydney Rosen[1,3], Bevis Phiri[4], Anna Grimsrud[5], Muya Mwansa[6], Hilda Shakwelele[4], Prudence Haimbe[4], Mpande M Mwenechanya[7], Priscilla Lumano Mulenga[6], Innocent Chiboma[6], Brooke E Nichols[1,2,3*]

[1]Health Economics and Epidemiology Research Office (HE^{2}RO), Department of Internal Medicine, Faculty of Health Sciences, University of the Witwatersrand, Johannesburg, South Africa; [2]Department of Medical Microbiology, Amsterdam University Medical Centre, Amsterdam, the Netherlands; [3]Department of Global Health, Boston University School of Public Health, Boston, MA, USA; [4]Clinton Health Access Initiative, Lusaka, Zambia; [5]International AIDS Society, Cape Town, South Africa; [6]Ministry of Health, Lusaka, Zambia [7]The Centre for Infectious Disease Research in Zambia, Lusaka, Zambia

Figure S1. Relative risk of loss to follow-up within 18 months of ART initiation for early enrollers of DSD models (ie. after <6 months of ART), stratified by dispensing period and age group (reference group: established enrollers of DSD models with >6 months of ART at DSD enrolment; analysis adjusted for sex and urban/rural status)

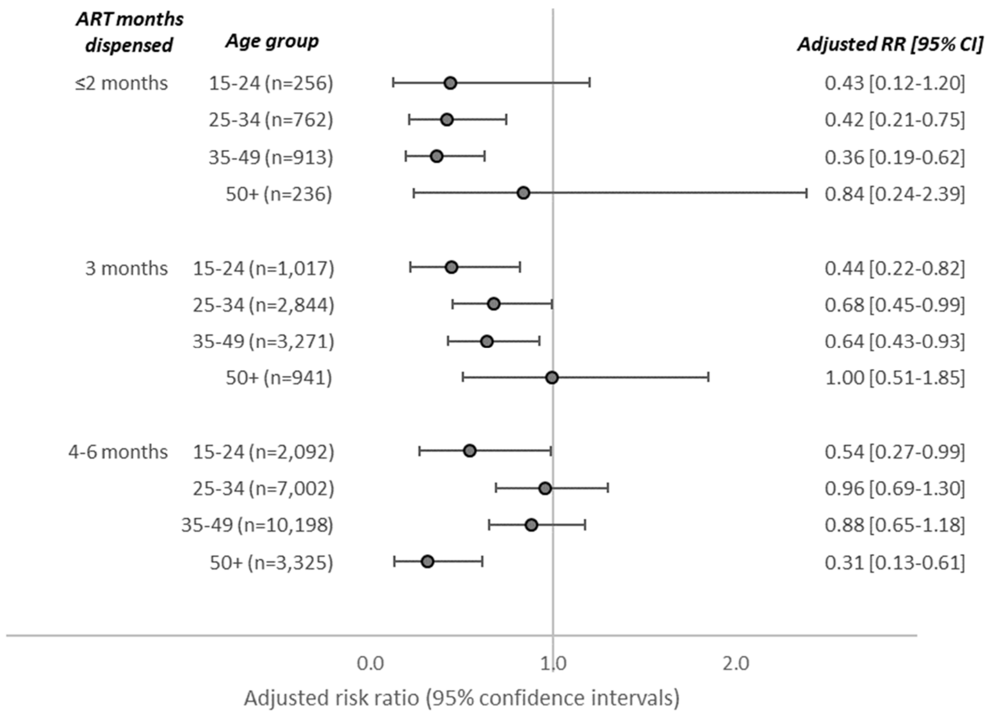

For peer review only - http://bmjopen.bmj.com/site/about/guidelines.xhtml

Figure S2. Proportion loss to follow-up by time on ART at DSD entry, limited to N=37 facilities with >20% of DSD patients at each facility classified as "early enrollers"

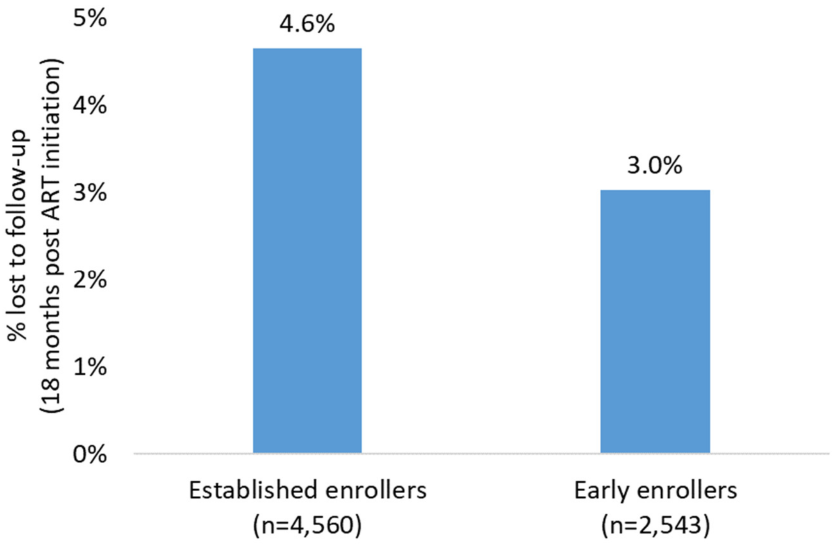

A potential area of concern was that facilities that had better-than-average retention would be more willing or able to enroll patients into DSD models early and therefore skew the results. We therefore conducted this sub-analysis where we limited the data to those facilities which had substantial proportion of their patients enrolled into DSD models early. Criteria for this analysis limited the data to facilities where: i) ≥20% of patients had early enrollers, and ii) at least 100 patients across both groups (early enrollers and established enrollers). 37 facilities across 8 of 10 provinces were selected for this analysis; 73% (n=27) of facilities were in urban areas. This analysis consisted of 7,103 patients: majority (61%, n=4,351) were female, age group distribution was similar to the main analysis (Table 2) (11%, n=784 were 15-24 years; 35%, n=2,488 were 25-34 years; 43%, n=3,028 were 35-49 years; 11%, n=799 were 50+ years), 81% (n=5,731) of patients were in urban settings. Majority (57%, n=4,028) of patients were enrolled into multi-month dispensing, 29% (n=2,058) were in fast-track, 7% (n=484) were in community pick-up points, 5% (n=350) were in home ART delivery, and <2% were in adherence groups (n=112) and extended clinic hours' groups (n=71).

Results show that in this subset of clinics, early enrollers were less likely to be lost to follow-up (3.0% [77/2,543]), compared to established enrollers (4.6% [212/4,560]). A log-binomial regression assessing risk of loss to follow-up, adjusting for age, sex, urban/rural status, and ART dispensing period estimated that, compared to established enrollers, early enrollers were 40% less likely to be lost to follow-up; adjusted risk ratios (aRR) 0.60 (95% CI 0.46-0.78).

For peer review only - http://bmjopen.bmj.com/site/about/guidelines.xhtml

STROBE Statement—Checklist of items that should be included in reports of ***cohort studies***

	Item No	Recommendation	Page No
**Title and abstract**	1	(*a*) Indicate the study's design with a commonly used term in the title or the abstract<br>(*b*) Provide in the abstract an informative and balanced summary of what was done and what was found	a, page 1; b, page 2
**Introduction**			
Background/rationale	2	Explain the scientific background and rationale for the investigation being reported	4-5
Objectives	3	State specific objectives, including any prespecified hypotheses	5
**Methods**			
Study design	4	Present key elements of study design early in the paper	5-6
Setting	5	Describe the setting, locations, and relevant dates, including periods of recruitment, exposure, follow-up, and data collection	5
Participants	6	(*a*) Give the eligibility criteria, and the sources and methods of selection of participants. Describe methods of follow-up<br>(*b*) For matched studies, give matching criteria and number of exposed and unexposed	5-6
Variables	7	Clearly define all outcomes, exposures, predictors, potential confounders, and effect modifiers. Give diagnostic criteria, if applicable	5-6
Data sources/ measurement	8*	For each variable of interest, give sources of data and details of methods of assessment (measurement). Describe comparability of assessment methods if there is more than one group	5-6
Bias	9	Describe any efforts to address potential sources of bias	5-6
Study size	10	Explain how the study size was arrived at	5-6
Quantitative variables	11	Explain how quantitative variables were handled in the analyses. If applicable, describe which groupings were chosen and why	5-7, Table 1
Statistical methods	12	(*a*) Describe all statistical methods, including those used to control for confounding<br>(*b*) Describe any methods used to examine subgroups and interactions<br>(*c*) Explain how missing data were addressed<br>(*d*) If applicable, explain how loss to follow-up was addressed<br>(*e*) Describe any sensitivity analyses	7
**Results**			
Participants	13*	(a) Report numbers of individuals at each stage of study—eg numbers potentially eligible, examined for eligibility, confirmed eligible, included in the study, completing follow-up, and analysed<br>(b) Give reasons for non-participation at each stage<br>(c) Consider use of a flow diagram	8, Figure 1
Descriptive data	14*	(a) Give characteristics of study participants (eg demographic, clinical, social) and information on exposures and potential confounders<br>(b) Indicate number of participants with missing data for each variable of interest<br>(c) Summarise follow-up time (eg, average and total amount)	8-9, Table 2
Outcome data	15*	Report numbers of outcome events or summary measures over time	9-10

For peer review only

For peer review only - http://bmjopen.bmj.com/site/about/guidelines.xhtml

Main results	16	(*a*) Give unadjusted estimates and, if applicable, confounder-adjusted estimates and their precision (eg, 95% confidence interval). Make clear which confounders were adjusted for and why they were included<br>(*b*) Report category boundaries when continuous variables were categorized<br>(*c*) If relevant, consider translating estimates of relative risk into absolute risk for a meaningful time period	10, Table 3
Other analyses	17	Report other analyses done—eg analyses of subgroups and interactions, and sensitivity analyses	11
**Discussion**			
Key results	18	Summarise key results with reference to study objectives	11
Limitations	19	Discuss limitations of the study, taking into account sources of potential bias or imprecision. Discuss both direction and magnitude of any potential bias	12-13
Interpretation	20	Give a cautious overall interpretation of results considering objectives, limitations, multiplicity of analyses, results from similar studies, and other relevant evidence	12-13
Generalisability	21	Discuss the generalisability (external validity) of the study results	13-14
**Other information**			
Funding	22	Give the source of funding and the role of the funders for the present study and, if applicable, for the original study on which the present article is based	14

*Give information separately for exposed and unexposed groups.

Note: An Explanation and Elaboration article discusses each checklist item and gives methodological background and published examples of transparent reporting. The STROBE checklist is best used in conjunction with this article (freely available on the Web sites of PLoS Medicine at http://www.plosmedicine.org/, Annals of Internal Medicine at http://www.annals.org/, and Epidemiology at http://www.epidem.com/). Information on the STROBE Initiative is available at http://www.strobe-statement.org.

For peer review only

For peer review only - http://bmjopen.bmj.com/site/about/guidelines.xhtml
